# Climate‐change‐driven shifts in C_3_ and C_4_ grass distributions and leaf traits could lead to changes in community‐level flammability

**DOI:** 10.1002/ajb2.70081

**Published:** 2025-08-08

**Authors:** Sarah L. Raubenheimer, Liting Zheng, Artur Stefanski, Peter B. Reich

**Affiliations:** ^1^ Institute for Global Change Biology, School for Environment and Sustainability University of Michigan Ann Arbor 48109, USA MI; ^2^ Department of Botany Rhodes University, Grahamstown South Africa; ^3^ Department of Forest Resources University of Minnesota St. Paul 55108, USA MN; ^4^ College of Natural Resources University of Wisconsin Stevens Point 54481 USA WI

**Keywords:** C_3_ grasses, C_4_ grasses, climate change, drought, elevated CO_2_, flammability, Great Plains, warming

## Abstract

**Premise:**

Climate change poses challenges to grasslands, including those of the North American Great Plains Region, where shifts in species distributions and fire dynamics are expected. Our present analysis focuses on remaining grasslands within this largely developed and agricultural region. The differential responses of C_4_ and C_3_ grass species to future climate conditions, particularly in habitat suitability and flammability, are critical for understanding ecosystem changes.

**Methods:**

We used species distribution models to predict shifts in habitat suitability for 37 grass species under future climate scenarios and assessed flammability traits in a free‐air CO_2_‐enrichment study, focusing on species' physiological responses to elevated CO_2_, warming, and drought.

**Results:**

Our models predicted that C_4_ species will retain higher habitat suitability, while C_3_ species will decline. Leaf‐level flammability analysis showed that species with higher water‐use efficiency under elevated CO will have lower flammability than under non‐elevated, potentially decreasing the predicted rate of fire spread when such species dominate. In contrast, species with higher growth rates but lower water‐use efficiency may be more flammable. Species‐specific responses varied within functional types. Anticipated shifts in species distributions suggest C_4_ species will become more dominant, potentially altering competitive dynamics and reducing C_3_ diversity. Changes in flammability under future conditions are expected to influence fire regimes, with a predicted decrease in mean community rate of spread due to the dominance of less‐flammable C_4_ species.

**Conclusions:**

These findings highlight the need for adaptive fire management and conservation strategies to maintain biodiversity and ecosystem function in North American grasslands under climate change.

Grasslands, such as those of the North American Great Plains Region (GPR), provide crucial ecological and economic benefits, including biodiversity, carbon sequestration, and agricultural productivity (Lark, 2020; Scholtz and Twidwell, 2022). Grasslands and savannas are experiencing declines in fire frequency, in contrast to patterns seen in forest ecosystems (Andela et al., 2017; Abatzoglou et al., 2018; Jones et al., 2022), altering ecosystem functioning, shifting species composition, and affecting flammability through changes in fuel loads and fire behavior. Anthropogenic factors, such as agricultural expansion, land‐use changes, and fire suppression, are recognized as primary drivers of these shifts (Archibald et al., 2013; Andela and Van Der Werf, 2014). However, there is growing interest in how climatic factors may influence fire regimes through changes to grassland community composition and species‐specific plant flammability (Manea et al., 2015; Raubenheimer et al., 2022; Havrilla et al., 2023). Fire regimes are influenced by both external factors, such as land‐use and climate change, and the inherent traits of vegetation. Therefore, understanding how plant flammability contributes to shifting fire dynamics is essential.

Plant flammability, a key determinant of fire activity globally (Bond and Van Wilgen, 1996; Beckage et al., 2009; Prior et al., 2017), is influenced by morphological and chemical plant traits that affect ignitability, sustainability of flaming, and combustibility (Rothermel, 1972). Climate‐change‐induced alterations in plant growth rates and leaf moisture content could significantly impact leaf ignitability, flaming intensity and time, and fire spread (Rothermel, 1972; Simpson et al., 2016; Gao and Schwilk, 2022; Nerlekar et al., 2025). The effects of climate change on leaf‐level flammability has been studied in Australia and southern Africa (Manea et al., 2015; Raubenheimer et al., 2022), but detailed analyses of climate‐driven changes to plant flammability in North American grassland communities remain notably scarce. Our present study addresses these gaps by investigating how increases in CO_2_ concentrations, temperature, nitrogen, and reductions in water availability influence grassland flammability in the North American context, focusing on changes to plant flammability and habitat suitability at the species and plant functional group (C_4_ and C_3_) level.

Climatic changes, such as rising CO₂ levels, increasing temperatures, and heightened aridity, are expected to significantly affect the leaf‐level flammability of grass species. Elevated CO_2_ (eCO_2_) can stimulate plant growth (Morgan et al., 2001; Zheng et al., 2018), increasing fire fuel load and potentially leading to more intense fires (Simpson et al., 2016). However, its impact on fuel moisture content is more nuanced. eCO_2_ improves water‐use efficiency (WUE) through reductions in stomatal conductance that decrease water loss through transpiration (Saralabai et al., 1997; Pastore et al., 2019). Heightened WUE can increase leaf moisture content, but the extent of this effect varies between C_3_ and C_4_ grasses and may be negligible in times of extreme drought. Increased temperatures create drier conditions and accelerate fuel leaf drying rates, influencing leaf ignitability and fire spread. C_4_ grasses generally tolerate water limitation and high temperatures better than C_3_ grasses and show greater enhancements to WUE under eCO_2_ (Hamim, 2005; Sage and Kubien, 2007; Wertin et al., 2015; Togawa‐Urakoshi and Ueno, 2022). Understanding these differences between C_3_ and C_4_ grasses—particularly in the context of the environmental conditions under which C_4_ photosynthesis evolved (Osborne et al., 2008)—is essential for predicting how future climate scenarios will alter fire regimes in grassland ecosystems.

As climate change progresses, the distributions of C_3_ and C_4_ grass species are likely to shift (Winslow et al., 2003; Pau et al., 2013; Anderson et al., 2024), impacting community composition and subsequently altering the overall flammability of grassland ecosystems. As temperatures rise and aridity increases, C_3_ grasses, which thrive in cooler, wetter conditions, may see range reductions and declines in abundance, while C_4_ grasses, adapted to warmer, drier environments, could expand their range and become more dominant (Ehleringer, 1978; Sage and Kubien, 2007; Havrilla et al., 2023; Anderson et al., 2024). Due to differences in photosynthetic pathways, C_3_ species have been expected to benefit from the rise of CO_2_ while C_4_ will not see much of the benefit; however, recent evidence suggests that C_4_ plants seem to thrive under eCO_2_ due to other mechanisms such as positive effects on soil N availability and heightened water use efficiency (Reich et al., 2018; Mohanbabu et al., 2024). Increased dominance of C_4_ species, which are also generally more tolerant of fire (Peterson et al., 2007; Moore et al., 2019), could lead to fire events causing further reductions in the dominance of C_3_ grasses.

In this study, we examined the impact of climate change on the flammability of grassland ecosystems by analyzing predicted shifts in species distributions and flammability‐related leaf traits in the Great Plains Region (GPR), with relevance limited to areas that remain as grassland. Using MaxEnt models, we projected changes in the distributions of 37 grass species characteristic of the GPR (21 C_4_ and 16 C_3_) from the present conditions to the 20‐year average predicted for the years 2041–2060 using the Model for Interdisciplinary Research on Climate Representative Concentration Pathway 8.5 (WorldClim climate prediction scenario MIROC6 SSP5 8.5 is shown in the results; additional scenarios are presented in the supplementary materials; Appendices S1–S3: Figures S1–S3) and estimated their future relative abundance within vegetation pixels across the region. For a subset of species, we predicted rates of fire spread using the Rothermel fire spread model (Rothermel, 1972), parameterized with leaf trait data from a long‐term free‐air CO_2_‐enrichment (FACE) experiment that investigates the effects of eCO_2_ (+CO_2_), elevated nitrogen (+N), elevated temperature (+T), and reduced rainfall (‐H_2_0) on mixed North American grassland communities. Our hypotheses were (1) C_4_ species will become more dominant as temperatures rise and aridity increases, while C_3_ species may shift to cooler, wetter regions; (2) C_4_ species will conserve more water under +CO_2_ (higher fuel moisture content) and increased productivity under combined +CO_2_, +N, +T, and ‐H_2_0, resulting in a higher fuel load than C_3_ species, but with water savings outweighing fuel load increases, leading to a reduced predicted rate of spread (ROS); and (3) the expected dominance of C_4_ species will likely lower the mean community‐level ROS in the GPR. By integrating species distribution models with physiological data from global change experiments, we aimed to refine predictions of future grassland community compositions and flammability, offering insights into how climate‐induced changes may alter fire dynamics in these vital ecosystems (Figure 1).

**Figure 1:**
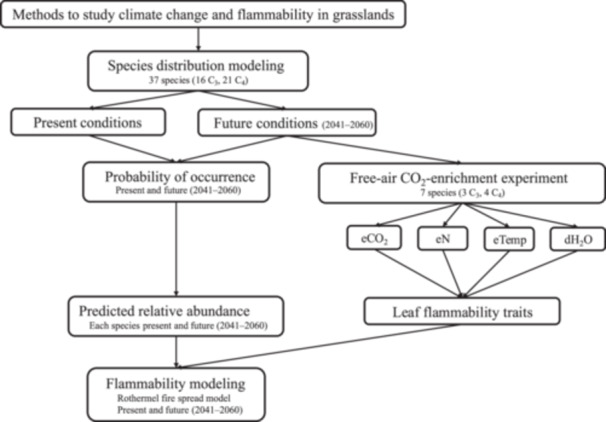
Conceptual diagram illustrating the methodological approach and integration of species distribution modeling, experimental trait data, and fire behavior modeling used in this study. MaxEnt models were developed for 37 grass species (21 C₄ and 16 C₃) characteristic of the Great Plains Region (GPR) under current and projected (2041–2060) conditions. These models provided estimates of habitat suitability and predicted relative proportion within the community. Concurrently, plant physiological traits related to flammability (for 3 C_3_ and 4 C_4_ species) were measured in a long‐term free‐air CO₂‐enrichment (FACE) experiment under elevated CO₂, nitrogen, temperature, and water limitation conditions. These trait data are used to parameterize the Rothermel fire spread model, enabling predictions of species‐specific and community‐level fire spread rates across the GPR under current and future scenarios.

## MATERIALS AND METHODS

### Species distribution modeling

Species distribution models (SDMs) were created using MaxEnt (v.3.3.3k) using the R package dismo: Species Distribution Modeling (Hijmans et al., 2023) for a selection of C_4_ and C_3_ grass species characteristic of the GPR (Table 1). MaxEnt applies machine learning to predict species presence or absence across geographic regions by analyzing the relationship between environmental conditions and species occurrence records (Elith et al., 2011). The model outputs a measure of habitat suitability (H.S.), which reflects both species density and the likelihood of presence (Elith et al., 2011). Despite the limitations of using presence‐only data (Phillips et al., 2009), MaxEnt SDMs provide a robust foundation for guiding physiological hypotheses (Valavi et al., 2022).

**Table 1:** List of species (including taxonomic authority and plant family) modeled with MaxEnt providing area under the curve (AUC) values for each species distribution model.

Metabolism type	Species	Authority	Family	AUC
C_4_	*Andropogon gerardii*	(Vitman) C.E. Hubb.	Poaceae	0.977
	*Andropogon hallii*	Hack.	Poaceae	0.988
	*Aristida purpurea*	Nutt.	Poaceae	0.984
	*Bouteloua curtipendula*	(Michx.) Torr.	Poaceae	0.98
	*Bouteloua dactyloides*	(Nutt.) J.T. Colt. & R.L. Slusher	Poaceae	0.993
	*Bouteloua gracilis*	(Willd. ex Kunth) Lag. ex Griffiths	Poaceae	0.978
	*Bouteloua hirsuta*	Lag.	Poaceae	0.987
	*Eragrostis trichodes*	(Nutt.) Alph. Wood	Poaceae	0.988
	*Hilaria jamesii*	(Torr.) Benth.	Poaceae	0.993
	*Muhlenbergia cuspidata*	(Torr. ex Hook.) Rydb.	Poaceae	0.984
	*Muhlenbergia filiformis*	(Thurb. ex S. Watson) Rydb.	Poaceae	0.993
	*Muhlenbergia reverchonii*	(Vasey) Rydb.	Poaceae	0.999
	*Panicum capillare*	L.	Poaceae	0.965
	*Panicum virgatum*	L.	Poaceae	0.985
	*Pappophorum bicolor*	E.Fourn.	Poaceae	0.996
	*Paspalum setaceum*	Michx.	Poaceae	0.977
	*Schizachyrium scoparium*	(Michx.) Nash	Poaceae	0.981
	*Sorghastrum nutans*	(L.) Nash	Poaceae	0.977
	*Sporobolus compositus*	(Poir.) Merr.	Poaceae	0.979
	*Sporobolus heterolepis*	(A. Gray) A. Gray	Poaceae	0.987
	*Tripsacum dactyloides*	(L.) L.	Poaceae	0.992
C_3_	*Achnatherum robustum*	(Vasey) Barkworth	Poaceae	0.995
	*Agropyron repens*	(L.) P.c Beauv.	Poaceae	0.956
	*Agrostis scabra*	Willd.	Poaceae	0.94
	*Alopecurus carolinianus*	Walt.	Poaceae	0.972
	*Bromus inermis*	Leyss.	Poaceae	0.971
	*Danthonia spicata*	(L.) P. Beauv. ex Roem. & Schult.	Poaceae	0.98
	*Elymus elymoides*	(Raf.) Swezey	Poaceae	0.982
	*Elymus villosus*	Muhl. ex Willd.	Poaceae	0.981
	*Festuca altaica*	Trin.	Poaceae	0.966
	*Hesperostipa comata*	(Trin. & Rupr.) Barkworth	Poaceae	0.979
	*Hesperostipa spartea*	(Trin.) Barkworth	Poaceae	0.989
	*Koeleria cristata*	(L.) Pers.	Poaceae	0.969
	*Nassella leucotricha*	(Trin. & Rupr.) R.W. Pohl	Poaceae	0.998
	*Nassella viridula*	(Trin.) Barkworth	Poaceae	0.982
	*Poa palustris*	L.	Poaceae	0.952
	*Poa pratensis*	L.	Poaceae	0.957

Species occurrence records were sourced from the Global Biodiversity Information Facility (GBIF.org, 2024; accessed 01 May 2024) and environmental data from WorldClim, providing 19 environmental layers for modeling (Table 2; http://www.worldclim.org; Fick and Hijmans, 2017) at a 30‐s resolution. Models were trained across the entire North American continent, yielding H.S. values on a 0 to 1 scale. To evaluate model performance, we implemented a random 50/50 split of the occurrence data for each species: 50% of the records were used to train the model, and the remaining 50% were used as an independent test set. The area under the curve (AUC) and additional evaluation metrics were calculated using this held‐out test set, in combination with 10,000 randomly sampled pseudoabsences. All models had AUC values above 0.95, with a mean AUC of 0.98 (Table 1).

**Table 2:** Description of environmental layers used in MaxEnt models. Environmental spatial data was obtained from the WorldClim Global Climate Data database (Fick and Hijmans, 2017).

Worldclim variable	Description
BIO1	Annual mean temperature
BIO2	Mean diurnal range [mean of monthly (max temp – min temp)]
BIO3	Isothermality (BIO2/BIO7)
BIO4	Temperature seasonality
BIO5	Max temperature of warmest month
BIO6	Min temperature of coldest month
BIO7	Temperature annual range (BIO5‐BIO6)
BIO8	Mean temperature of wettest quarter
BIO9	Mean temperature of driest quarter
BIO10	Mean temperature of warmest quarter
BIO11	Mean temperature of coldest quarter
BIO12	Annual precipitation
BIO13	Precipitation of wettest month
BIO14	Precipitation of driest month
BIO15	Precipitation seasonality (coefficient of variation)
BIO16	Precipitation of wettest quarter
BIO17	Precipitation of driest quarter
BIO18	Precipitation of warmest quarter
BIO19	Precipitation of coldest quarter

Habitat suitability was predicted for each species in the present conditions and projected future conditions (20‐year average for 2041–2060). Results are shown in the main text for the MIROC6 model under scenario SSP5‐8.5; additional models and scenarios are included in the supplementary appendices (MIROC6, Appendix S4: Figure S4a and b; ACCESS‐CM2, Appendix S1: Figure S1a and b; CMCC‐ESM‐2‐0, Appendix S2: Figure S2a and b; EC‐Earth3‐Veg, Appendix S3: Figure S3a and b; SSP3‐7.0 and SSP5‐8.5). Across models within each scenario, predicted changes in habitat suitability were highly correlated (Pearson's *r* ≥ 0.80, typically >0.90), justifying the use of MIROC6 SSP5‐8.5 as representative (Appendix S5: Table S5).

### Relative proportion estimates

The SDM outputs provided H.S. measures for 37 species across North America. The relative proportion of each species within a model plant “macrocommunity” (i.e., within the regional vegetation) in the GPR was calculated for present and future conditions by summing the modeled probabilities of all species within each 30‐s grid cell:

(1) $$P_{j}=\sum_{i=1}^{37}p_{\mathrm{ij}},$$

where *P*_*j*_ is the total probability of occurrence at location *j*, *p*_*ij*_ is the probability of occurrence for species *i* at location *j*. This sum represents the total probability of species occurrence at a location. The predicted relative proportion of each species *i* was then calculated as the fraction of the community likely to be represented by that species at a given location *j*:

(2) $$j=\frac{p_{\mathrm{ij}}}{P_{j}}.$$

The summation was carried out across all 37 species.

### Experimental setup and flammability‐related leaf traits measurements

Data were collected between mid June to mid July at the BioCON and TeRaCON experiments at Cedar Creek Ecosystem Science Reserve, Minnesota, United States (Reich et al., 2001, 2014, 2020). We focused on seven grass species, including four C_4_ species (*Andropogon gerardii*, *Bouteloua gracilis*, *Schizachyrium scoparium*, *Sorghastrum nutans*) and three C_3_ species (*Agropyron repens*, *Bromus inermis*, *Koeleria cristata*). The experiments encompassed a range of environmental treatments in an incomplete factorial design, including ambient conditions, eCO_2_ (+CO_2_), elevated nitrogen (+N), elevated temperature (+T), reduced water availability (‐H_2_O), and biodiversity treatments (monocultures, and 4‐, 9‐, and 16‐species mixtures). For this analysis, we focused on the 9‐species mixtures because they enable testing of joint CO_2_, N, water availability and temperature treatments (Reich et al 2020). The species in these mixtures were randomly assigned from a pool of 16 native or naturalized species at the site, comprising C_3_ and C_4_ grasses, legumes, and non‐leguminous forbs. Further details on species selection and experimental design were provided by Reich et al. (2001).

Plant biomass per unit ground area and height were estimated through destructive and non‐destructive methods in June‐July 2023. Aboveground biomass was estimated by clipping and collecting a single 10 × 100 cm strip just above the soil surface from each plot in July 2023. Leaf traits, including water content and specific leaf area, were measured in June 2023 (Table 3). Species and treatments were randomly selected daily to minimize sampling bias. Gas exchange measurements were conducted in June 2023, on warm, sunny days (25–30°C, photosynthetically active radiation [PAR] >1500 μmol m² s⁻¹) to ensure consistent conditions, using a LI‐COR 6400 portable photosynthesis system (LI‐COR Biosciences, Lincoln, NE, USA). Leaf‐to‐air vapor pressure deficit (<1.5 kPa) and block temperature (25°C) were controlled during measurements. Photosynthetically active radiation was set at 1500 μmol m² s⁻¹, and reference CO_2_ concentrations were maintained at 400 ppm and 550 ppm to evaluate potential downregulation of photosynthesis under eCO_2_. Carbon assimilation rate (*A*), stomatal conductance (*G*_st_), transpiration rate (*E*), and WUE (*A*/*E*) were recorded for each treatment combination (*N* = 8 per treatment).

**Table 3:** Summary of plant leaf traits and their hypothesized impacts on flammability.

Trait	Abbreviation	Unit	Impact on flammability
Specific leaf area	SLA	cm² g	A higher SLA may increase flammability and fire spread rate due to thinner leaves, which lower heat capacity and provide a larger surface area for rapid heat absorption and moisture evaporation (Burton et al., 2021). Thinner leaves also dry out faster during fire season, likely leading to lower LWC.
Leaf water content	LWC	%	Higher LWC generally reduces flammability because moist leaves require more energy to heat and evaporate moisture, making them slower to ignite (Rothermel, 1972; Simpson et al., 2016). The evaporating water also cools the leaf surface, further delaying ignition. High LWC significantly impacts community‐level fire regimes (Cardoso et al., 2018; Zubkova et al., 2019).
Water‐use efficiency	WUE	µmol CO₂ mmol H₂O	Greater WUE might correlate with slower moisture loss, potentially reducing flammability (Raubenheimer et al., 2022).
Stomatal conductance	*G*_st_	µmol H₂O m⁻² s⁻¹	Lower *G*_st_ could maintain higher moisture levels in leaves, decreasing flammability through higher LWC.
Carbon assimilation rate	*A*	µmol CO₂ m⁻² s⁻¹	Higher rates of *A* might indicate more robust growth, potentially leading to denser foliage that could affect fire behavior (Raubenheimer et al., 2022). If higher *A* leads to increases in biomass, flammability could be increased.
Aboveground biomass	AG biomass	g	Larger biomass can contribute to higher fuel loads, thus potentially increasing flammability, although this can be countered by high moisture content (Simpson et al., 2016; Raubenheimer et al., 2022).

### Principal component analysis (PCA) and clustering

To investigate the underlying structure of the leaf trait data, we performed a PCA using the R package FactorMineR (Lê et al., 2008). The PCAs were conducted for all species pooled but visualized by functional type. The analysis revealed nine principal components (PCs), with the first five explaining 36.6%, 17.8%, 13.4%, 10.7%, and 10.2% of the total variance, respectively. The corresponding eigenvalues for these components were 3.29, 1.61, 1.21, 0.96, and 0.92, indicating that the first few components captured the majority of the variance. Subsequently, a *k*‐means clustering analysis was conducted, identifying three distinct clusters within the data. The clusters contained 364, 222, and 164 observations, respectively. The cluster centroids in the first two PCA dimensions were: Cluster 1 with mean values of –1.10 (PC1) and 0.70 (PC2), Cluster 2 with mean values of –0.25 (PC1) and –1.41 (PC2), and Cluster 3 with mean values of 2.78 (PC1) and 0.35 (PC2).

### Flammability modeling

To assess species‐specific flammability impacts under experimental treatments in the GPR, we applied the Rothermel fire model (Rothermel, 1972) to predict the rate of fire spread (ROS). This analysis utilized the function rothermel in the R package firebehavioR (Ziegler, 2019) in R version 4.4.1 (R Core Team, 2024). We parameterized the model for each species, assuming continuous grass layers and aligning with the specified D fuel model type, which represents “Southern Rough” fuels—characterized by a mix of shrubs, hardwood litter, and grassy understory with a fuel bed depth of approximately 1.5 feet and a moisture of extinction near 20%. Parameterization included live biomass from our experimental data and maximum dead biomass and heat levels as described in the reference manual for the R package firebehavioR (Ziegler, 2019). Fuel moisture was defined using our experimental measurements of leaf water content. Environmental conditions were used for both present and future (2060, MIROC SSP5 8.5) scenarios. We prepared a 10‐m‐resolution gridded data set covering necessary variables: slope, wind speed, and soil moisture. Slope was derived from NASA SRTM data (NASA JPL, 2013) using the terrain function in R. Wind data extracted from the Wind Integration National Dataset (WIND) Toolkit (Draxl et al., 2015). Soil moisture was calculated based on precipitation data from the CHELSA database (Karger et al., 2017), incorporating wilting points and field capacities from the IGBP Global Soil Data Task (Global Soil Data Task, 2000) as described by Trabucco and Zomer (2010). This was correlated with plant moisture content through linear models developed during our study. All environmental data layers were synchronized in extent and resolution via the R package raster (Hijmans, 2023), facilitating species and treatment‐specific fire behavior modeling across all grid cells. Community levels of ROS incorporated the species‐specific modeled ROS weighted by the predicted relative abundance of each species within the community (as calculated in Equations 1 and 2).

### Statistical analyses

We conducted a series of statistical analyses using linear mixed‐effects models (LMMs) implemented through the R package lme4 (Bates et al., 2015). These models were used to evaluate the effects of plant type (C_3_ vs. C_4_), environmental scenarios (ambient vs. future), and their interactions, with random effects included to account for species and spatial variability. For H.S., LMMs were employed to compare the effects of plant type and scenario across North America, including post hoc pairwise comparisons to examine specific differences between combinations of plant types and scenarios (Appendix S6: Table S6a, b). Additionally, linear models were used to assess scenario effects on H.S. for each species individually (Appendix S7: Table S7). In the analysis of relative community composition within the GPR, LMMs were used to test the main effects of plant type and scenario, as well as their interaction, with species treated as a random effect to account for species variability (Appendix S8: Table S8a and b). To evaluate predicted rates of fire spread using the Rothermel fire model, we used LMMs to compare current conditions with two future scenarios, incorporating random effects for spatial coordinates to account for spatial heterogeneity (Appendix S9: Table S9a). Estimated marginal means (EMM) were computed for each species under different scenarios, and pairwise comparisons were used to identify significant differences in species responses to future environmental conditions (Appendix S9: Table S9b). This suite of analyses allowed us to rigorously test our hypotheses regarding plant and community responses to changing environmental conditions.

## RESULTS

### Shifts in habitat suitability with climate change

**Figure 2:**
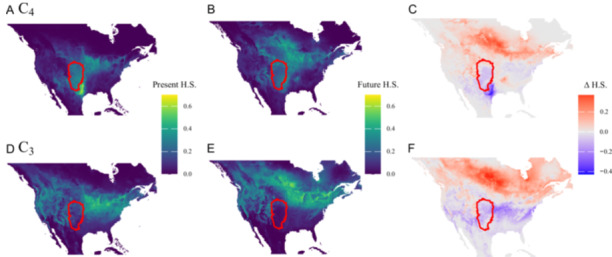
Changes in habitat suitability (H.S.) for C_4_ (top row) and C_3_ (bottom row) grass species characteristic of the Great Plains Region (GPR). Panels A and D represent present conditions, while B and E depict projected conditions based on the 20‐year average climate predicted for the years 2041–2060 (MIROC6 SSP5 8.5; additional scenarios are presented in Appendices S2–S4). Panels C and F show the difference between future and present suitability (∆H.S.). The red outline indicates the boundaries of the Great Plains Region (GPR).

Species distribution models (SDMs) indicated potential shifts in mean habitat suitability (H.S.) for C_3_ and C_4_ grass species characteristic of the GPR (red outlines in Figure 2) as the climate changes from present conditions (Figure 1A, D) to future projections for 2060 (MIROC6 SSP5 8.5; Figure 2B, E). See Appendix S10: Figure S10 for a summary of the relationship between mean predicted H.S. relative to bioclimatic variables. The values presented are the mean predictions for 37 selected species, with individual species models available in Appendix S11 (Figure S11) for C_4_ species and Appendix S12 (Figure S12) for C_3_ species.

Under the present environmental conditions, the SDMs predicted that C_4_ species (Figure 2A) generally had higher mean H.S. than C_3_ species (Figure 1D) within the GPR (mean suitability: C_4_ = 0.23, C_3_ = 0.13; Est. = –0.10, SE = 0.04, *z* = –2.94, *P* < 0.05; see Table S1). However, both C_4_ and C_3_ species had some level of suitability, suggesting their potential presence in the region. In future scenarios, C_4_ species maintained relatively high average H.S. (Figure 1B; mean = 0.20), with increases in the northern GPR and decreases in the southern GPR (Figure 2C). In contrast, C_3_ species are projected to have lower average habitat suitability (H.S.; Figure 2E; mean = 0.08) compared to C_4_ species, with predicted distributions showing both stable and declining areas across the region (Figure 2C). The significant interaction between plant type and scenario underscores the divergent responses of C_3_ and C_4_ species to future climatic conditions (Est. = 0.02, SE = 0.001, *t* = 16.60, *P* < 0.0001; see Table S2), with C_4_ species having a smaller reduction in habitat suitability compared to C_3_ species (Est. for C_3_ future vs. C_4_ future = –0.12, SE = 0.04, *z* = –3.44, *P* < 0.01; see Table S2).

### Shifts in the mean predicted relative proportion of plant communities

**Figure 3:**
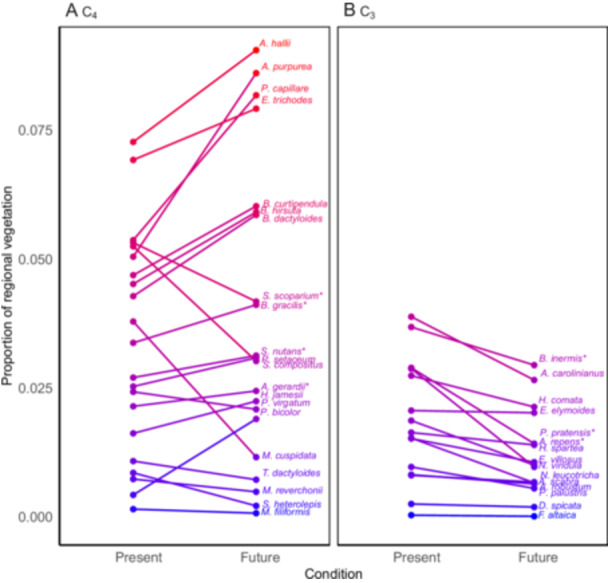
Predicted changes in the mean relative proportion of (A) C_4_ and (B) C_3_ grass species within the vegetation of the Great Plains Region (GPR). These projections compare present conditions to the 20‐year average predicted for the years 2041–2060 based on the MIROC6 SSP5 8.5 climate scenario (additional scenarios are presented in Appendices S2–S4). Species with an asterisk were used in the leaf trait analysis and flammability modeling shown in Figures 4 and 5. Red denotes species with higher predicted proportions, while blue indicates species with lower predicted proportions.

Figure 3 depicts changes in the predicted mean relative proportion of C_4_ (Figure 3A) and C_3_ (Figure 3B) grass species within the modeled vegetation in the GPR, comparing present conditions with projections for the 20‐year average predicted for the years 2041–2060 (MIROC6 SSP5 8.5).

Under the present conditions, the mean predicted relative proportion of each species within the plant region was similar for C_3_ and C_4_ species (Est. = –0.02, SE = 0.01, *z* = –2.42, p = 0.09). However, under future environmental conditions, C_4_ species were projected to comprise a significantly greater proportion of the community compared to C_3_ species (Est. = 0.02, SE = 0.01, *z* = 3.17, *P* < 0.05). Specifically, 57% of C_4_ species were predicted to increase their relative proportion within the region, 24% expected to maintain their current proportion, and 19% anticipated to show decreases. Conversely, all C_3_ species were projected to either decrease (66%) or maintain (33%) their relative proportion within the regional vegetation (Est. = –0.02, SE = 0.01, *z* = –2.96, *P* < 0.05). The species predicted to show the most significant increases in relative proportion were, not surprisingly, those tolerant of dry, sandy soils and known to recover successfully after fire.

### Plant leaf traits under different global change treatments

**Figure 4:**
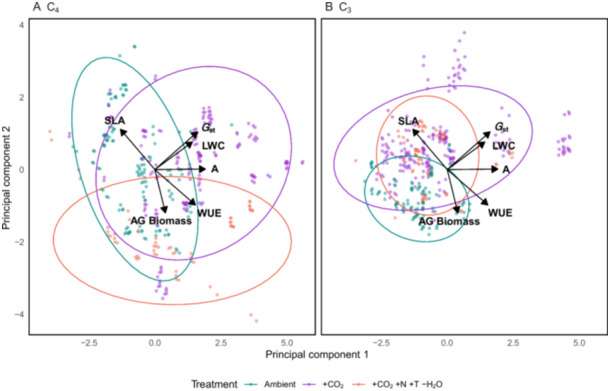
Principal component analysis (PCA) of plant traits done for all species pooled but visually faceted by (A) C_3_ and (B) C_4_ plant type in different treatments (ambient, +CO_2_, and +CO_2_ + N + T ‐H_2_O). The figure displays the distribution of individual plant samples based on the first two principal components (PC1 and PC2). Trait vectors are shown as arrows with labels indicating the traits: specific leaf area (SLA; cm^2^ g), leaf water content (LWC; %), aboveground biomass (AG Biomass; g), photosynthetic rate (*A*; µmol CO_2_ m^–2^ s^–1^), stomatal conductance (*G*_st_; µmol H_2_O m^–2^ s^–1^), and water–use efficiency (WUE; µmol CO_2_ mmol H₂O). The PCA shows the multivariate trait responses of C_3_ and C_4_ species in different treatments, with ellipses representing 95% confidence intervals around the multivariate means.

We analyzed the effects of eCO_2_ and combined environmental treatments (+CO_2_, +N, +T, ‐H_2_O) on various traits in a subset of C_4_ (*Andropogon gerardii*, *Bouteloua gracilis*, *Schizachyrium scoparium*, *Sorghastrum nutans*) and C_3_ (*Agropyron repens*, *Bromus inermis*, *Koeleria cristata*) grass species grown in mixture. Our linear mixed‐effects models (Appendix S13: Table S13) revealed significant treatment effects and species–functional‐type interactions on several traits. The separation between treatments was more pronounced in C_4_ species (Figure 4A), indicating greater variability in trait responses compared to C_3_ species (Figure 4B). The separation between treatments in C_4_ species (Figure 4A) reflected strong increases in aboveground biomass under the combined treatment (interaction Est. = 119.32, SE = 5.44, *t* = 21.95, *P* < 0.0001), with no significant effects on gas exchange, leaf water content, or WUE. In contrast, C_3_ species experienced significant declines in biomass under both eCO_2_ (Est. = −17.26, SE = 3.89, *t* = −4.44, *P* < 0.01) and the combined treatment (Est. = −29.41, SE = 6.00, *t* = −4.90, *p* < 0.01), alongside a marginal increase in SLA (Est. = 39.00, SE = 17.4, *t* = 2.24, *P* = 0.06). Overall, the data highlighted divergent biomass responses between functional types under global change treatments, with limited evidence for treatment effects on underlying physiological traits.

### Changes in the mean predicted rate of fire spread (ROS) under different environmental scenarios

**Figure 5:**
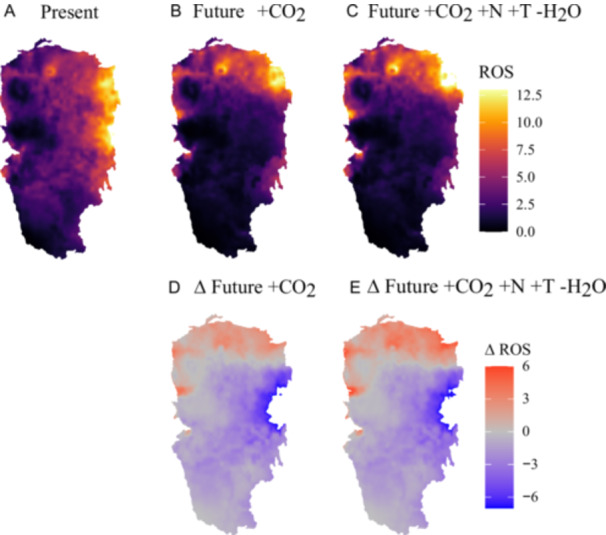
Changes in the mean predicted rate of fire spread (ROS) for a plant community consisting of eight grass species from the BioCON experiment (indicated by an asterisk in Figure 3) within the Great Plains Region (GPR). Panel A represents present conditions; panel B shows future projections for the 20‐year average for the years 2041–2060 (MIROC SSP5 8.5; additional scenarios are presented in the supplementary materials) incorporating the effects of elevated CO_2_ (+CO_2_) on flammability‐related leaf traits. Panel C further includes the effects of elevated nitrogen (+N), elevated temperature (+T), and reduced water availability (‐H_2_O) on flammability‐related leaf traits. Panels D and E illustrate the differences in ROS between ambient conditions and future scenarios incorporating (D) elevated CO_2_ and (E) combined effects of elevated CO_2_, nitrogen, temperature, and reduced water. Mean ROS is weighted by the predicted relative proportion of each species as calculated from the species distribution models shown in Figures 2 and 3. Species included in ROS models are *Andropogon gerardii*, *Schizachyrium scoparium*, *Sorghastrum nutans* (C_4_ grass species), *Agropyron repens*, *Bromus inermis*, and *Koeleria cristata* (C_3_ grass species). Predicted ROS under different climate scenarios for each species is shown in Appendix S14.

Figure 5 shows the predicted changes in the ROS for mixed grass communities across the GPR based on measured leaf traits under climate change treatments shown in Figure 4, comparing current conditions (Figure 5A) with two future scenarios: Future environmental conditions incorporating the effects of eCO_2_ (+CO_2_) on flammability‐related leaf traits (Figure 5B), and future environmental conditions that include in addition the effects of elevated nitrogen (+N), elevated temperature (+T), and reduced water availability (‐H_2_0) on flammability‐related leaf traits (Figure 5C). ROS predictions were weighted by the relative proportion of each species in the community, based on the predicted estimates shown in Figure 3. Predicted ROS under different climate scenarios for each species is shown in Appendix S14: Figure S14 and Table S14.

Under present conditions, the mean ROS for the GPR was estimated at 42.09, although there is much geographical variation because conditions differ (Figure 5). Although there are increases in ROS predicted for the northern regions of the GPR, significant reductions in ROS were projected in the central and southern regions for both future scenarios. Specifically, the Future +CO_2_ scenario showed a mean decrease of 5.38 units compared to present conditions (Est. = –5.38, SE = 0.01, *t* = –468.37, *P* < 0.0001), while Future +CO_2_, +N, +T and ‐H_2_0 showed a mean decrease of 2.48 units (Est. = –2.48, SE = 0.01, *t* = –215.87, *P* < 0.0001). Figure 4 highlights the differences between present and future scenarios. The reduction in ROS was greater when comparing present conditions to Future +CO_2_ than when comparing present conditions to Future +CO_2_, +N, +T and ‐H_2_0. The additional effects in Future +CO_2_, +N, +T and ‐H_2_0 further reduced ROS compared to Future +CO_2_ (Est. = –2.90, SE = 0.01, *z* = –252.50, *P* < 0.0001; Appendix S9). See Appendix S14 (Figure S14, Table S14) for species‐specific responses.

## DISCUSSION

The predicted shifts in habitat suitability, plant community composition, and fire dynamics within the GPR highlight the profound ecological impacts that climate change may impose on grassland ecosystems. This study aimed to assess how C_4_ and C_3_ grass species, which are integral to the GPR, will respond to the 20‐year average climate conditions predicted for the year range 2041–2060. Our findings reveal significant differences in the way these plant types will adapt to changing environments, with C_4_ species generally maintaining higher habitat suitability and potentially increasing their dominance within plant communities. Additionally, the predicted reduction in the rate of fire spread (ROS), linked to plant physiological responses to eCO_2_, under future scenarios suggests alterations in fire regimes that could further influence ecosystem structure and function.

**Figure 6:**
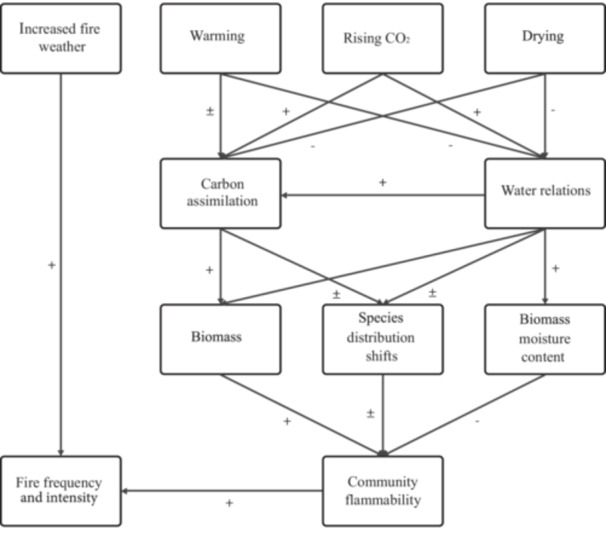
Conceptual model illustrating the interactions between climate change factors and plant physiological responses. The diagram depicts the direct and indirect impacts of increased fire weather, warming, rising CO_2_ levels, and drying on plant traits such as photosynthetic capacity, biomass, and moisture content. These traits subsequently influence larger ecological dynamics such as species distribution shifts, fire frequency and intensity, and community flammability. +, positive impacts; –, negative impacts; ±, variable impacts.

The habitat suitability models for C_4_ and C_3_ species indicate a significant divergence in their responses to future climate scenarios, despite variability within the groups. (See Figure 6 for a conceptual model illustrating the interactions between climate change factors and plant physiological responses.) C_4_ species were in general predicted to retain relatively high habitat suitability across much of the GPR, particularly in its northern regions, whereas C_3_ species tended to show a pronounced decline in suitability. The species that showed the most significant increases (e.g., *A. hallii*, *A. pupurea*, *A. gerardii*) in habitat suitability into the future are all currently well adapted to dry, sandy soils (Uchytil, 1988; USDA‐NRCS Plant Materials, 2015; Falk et al., 2020), supporting our model's suggestion that they would be more tolerant of the warmer, drier conditions expected in the future. These trends are consistent with the known physiological advantages of C_4_ plants in warmer, drier conditions because they possess a more efficient carbon fixation pathway and greater water‐use efficiency compared to C_3_ species (Ehleringer, 1978; Sage and Kubien, 2007; Pau et al., 2013; Yamori et al., 2014). The trends are also consistent with the competitive advantage shown by C_4_ grasses, and in particular, *A. gerardii* (C_4_), under long‐term ecologically realistic settings under eCO_2_, that likely involve species interactions and plant–soil feedbacks (Reich et al., 2018; Mohanbabu et al., 2024). These shifts underscore the importance of considering both plant physiological and ecological responses when predicting species responses to climate change and suggest potential decreases in biodiversity within the GPR.

The projected increase in the relative proportion of C_4_ species within the GPR's plant communities is not surprising (other than perhaps with respect to eCO_2_) yet has broad ecological implications (Palmquist et al., 2021; Havrilla et al., 2023), some of which might be surprising. C_4_ species have been widely expected to become more dominant as the climate changes, although studies have found the opposite response in some species sets (Knapp et al., 2020) and questions about the control of soil conditions on range expansion have been raised (Havrilla et al., 2023). Shifts toward C_4_ dominance could alter competitive dynamics within plant communities, potentially leading to further decreases in the diversity of C_3_ species because they may become outcompeted by the dominant C_4_ species. The dominance of C_4_ species could also impact ecosystem functions such as nutrient cycling, primary productivity, and palatability of forage for livestock (Barbehenn et al., 2004). The traits that enable C_4_ species to thrive under future conditions—such as resilience to fire, heat, and drought—will likely shape the structure and function of the GPR's grasslands in the coming decades.

The predicted species‐level ROS reveals notable variability under future climate scenarios, with no clear difference between C_4_ and C_3_ species. Some species, particularly those with traits favoring high water‐use efficiency, are expected to experience significant reductions in ROS. For instance, species like *A. gerardii* (C_4_) and *A. repens* (C_3_) may see a pronounced decrease in ROS due to their ability to maintain higher leaf moisture under eCO_2_ conditions. Conversely, species that respond to eCO_2_ with significant growth increases but lower water savings, such as *S. scoparium* (C_4_), may exhibit increases in ROS. These species‐specific responses suggest that future fire dynamics within the GPR will not only depend on the broader environmental context but also on the unique physiological traits of the most dominant species. Additionally, the predicted reduction in ROS in the future, linked to plant physiological responses to eCO_2_, under future scenarios suggests possible alterations in fire regimes that could further influence ecosystem structure and function (see Figure 6).

The general reduction in the mean community predicted ROS under future climate scenarios is a notable outcome of this study. Although our models predict increases in ROS in the northern regions of the GPR, we predict a general decrease in ROS over the entire region. The ROS is expected to decrease more significantly under scenarios incorporating the effects of eCO_2_ on plant physiology, which likely reduce plant flammability by altering leaf moisture in the species predicted to be more dominant. This trend could lead to less frequent and less intense fires in the GPR, which in turn might influence species composition and the overall resilience of grassland ecosystems. The comparison between the effects of eCO_2_ alone and the combined effects of eCO_2_, elevated N, elevated temperature, and reduced water availability provides valuable insights into how different environmental factors might interact to shape future fire regimes.

Our projections of shifts in community composition and flammability are uncertain because each step in our analyses contains considerable uncertainty. Nonetheless, the findings of this study have important implications for conservation and restoration efforts in the GPR. Because C_4_ species are likely to become more dominant, conservation strategies may need to focus on preserving the remaining habitats suitable for C_3_ species to maintain biodiversity. These shifts underscore the importance of considering both plant physiological and ecological responses when predicting species responses to climate change and suggest potential decreases in biodiversity within the GPR (Figure 6). Additionally, the somewhat counter‐intuitive predicted changes in flammability suggest that restoration efforts may need to incorporate fire management practices that account for increased flammability and predicted fire spread rates in the northern GPR and reduced fire spread in the central and southern GPR. Future research should aim to validate these predictions through long‐term monitoring and experimental studies, particularly those that can further explore the complex interactions between climate change, plant physiology, and fire ecology. Such work would advance initial steps, such as done herein, which contain considerable uncertainty. Understanding these dynamics will be crucial for developing adaptive management strategies that can mitigate the impacts of climate change on the GPR's grasslands.

This study provides valuable insights into the potential ecological shifts within the remaining grasslands of the GPR as a result of climate change. The predicted resilience of C_4_ species and the decline of C_3_ species highlight the need for targeted conservation efforts to preserve biodiversity in these grasslands. Additionally, the anticipated changes in fire dynamics will require adaptive management approaches to maintain the ecological functions of these ecosystems. As the climate continues to change, understanding and preparing for these shifts will be essential for the long‐term conservation and management of the GPR.

## AUTHOR CONTRIBUTIONS

S.L.R. and P.B.R. conceived the ideas and designed the methodology; S.L.R, L.Z., and A.S. collected the data, S.L.R. analyzed the data and led the writing of the manuscript. All authors contributed critically to the manuscript and gave final approval for publication.

## Supporting information


**Appendix S1.** Change in habitat suitability predicted for 2060 using ACCESS CM‐2.

**Appendix S2.** Change in habitat suitability predicted for 2060 using CMCC‐ESM‐2‐0.

**Appendix S3.** Change in habitat suitability predicted for 2060 using EC‐Earth3‐Veg.

**Appendix S4.** Change in habitat suitability predicted for 2060 using MIROC6.

**Appendix S5.** Pairwise Pearson correlation coefficients between habitat suitability projections across GCMs.

**Appendix S6.** Habitat suitability: summary of linear mixed‐effects model results for C_4_ and C_3_ species.

**Appendix S7.** Species‐specific habitat suitability: summary of linear model results.

**Appendix S8.** Community composition: summary of linear mixed‐effects model results for C_4_ and C_3_ species.

**Appendix S9.** Predicted rate of fire spread: summary of linear mixed‐effects model results.

**Appendix S10.** Habitat suitability: effect of bioclimatic variables (C_3_ and C_4_).

**Appendix S11.** Species‐specific habitat suitability (C_4_).

**Appendix S12.** Species‐specific habitat suitability (C_3_).

**Appendix S13.** Traits: summary of linear mixed‐effects model results.

**Appendix S14.** Species‐specific predicted rate of fire spread.

## Data Availability

Data are available on the Dryad Digital Repository (https://doi.org/10.5061/dryad.dv41ns29n).
