# Supplementary material for: Climate‐change‐driven shifts in C3 and C4 grass distributions and leaf traits could lead to changes in community‐level flammability
Source: Am J Bot. 2025 Aug 8;112(10):e70081. doi: 10.1002/ajb2.70081 (PMC12572686; doi:10.1002/ajb2.70081)
Supplement: Supplementary file 4 — Appendix S4. Change in habitat suitability predicted for 2060 using MIROC6. [file AJB2-112-e70081-s012.pdf]

Appendix S4. Change in habitat suitability predicted for 2060 using MIROC6.

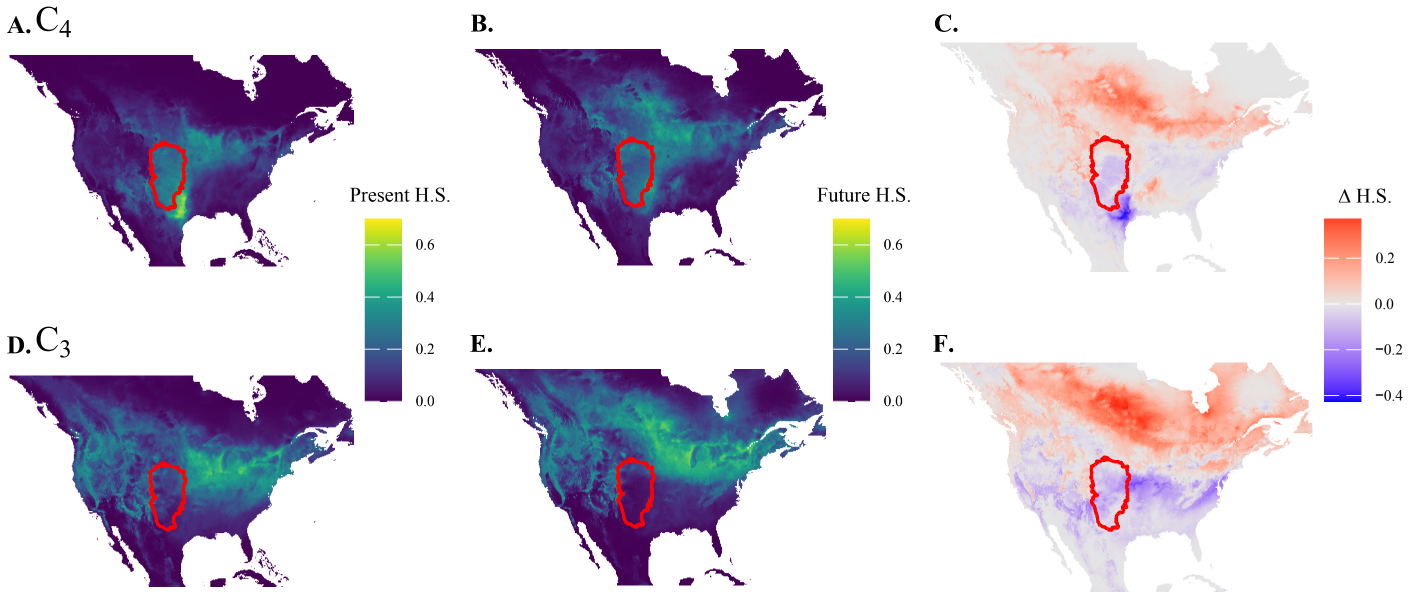

Figure S4a. Changes in habitat suitability (H.S.) for C_4 (top row) and C_3 (bottom row) grass species characteristic of the Great Plains Region (GPR). Panels A and D represent present conditions; B and E depict projected conditions based on the 20-year average climate predicted for the years 2041–2060 (MIROC6 SSP3 7.0). Panels C and F show the difference between future and present suitability (ΔH.S.). The red outline indicates the boundaries of the Great Plains Region (GPR).

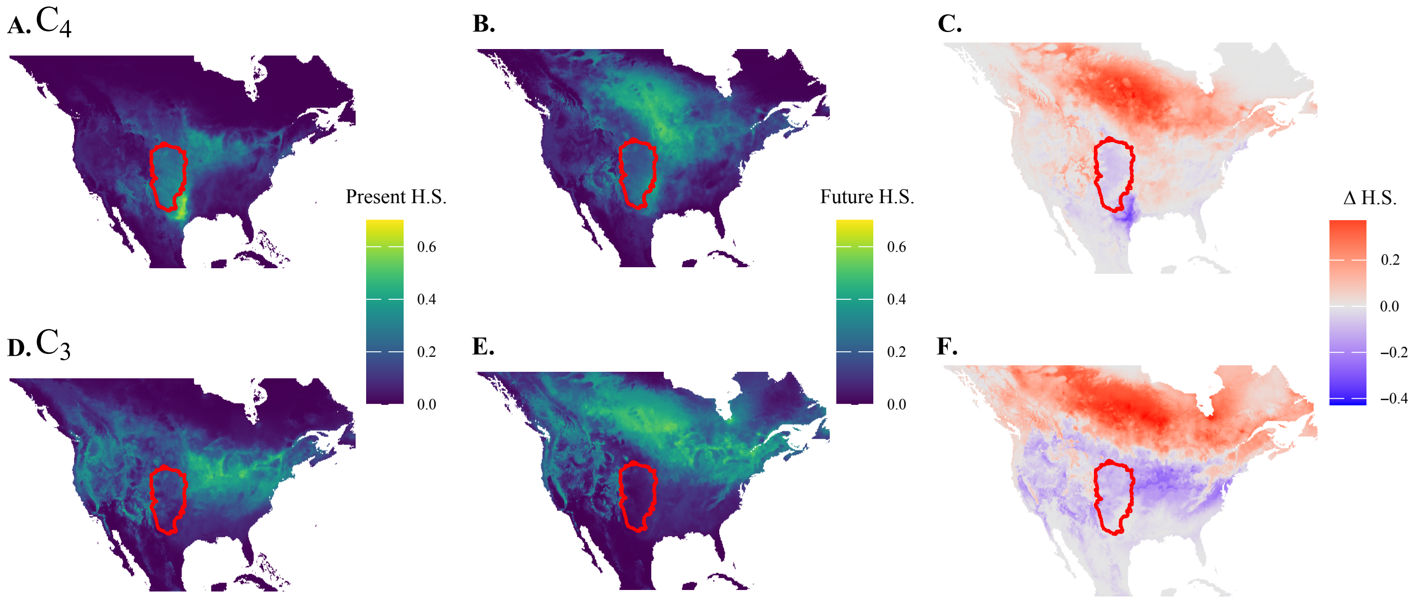

Figure S4b. Changes in habitat suitability (H.S.) for C_4 (top row) and C_3 (bottom row) grass species characteristic of the Great Plains Region (GPR). Panels A and D represent present conditions; B and

E depict projected conditions based on the 20-year average climate predicted for the years 2041-2060 (MIROC6 SSP5 8.5). Panels C and F show the difference between future and present suitability (ΔH.S.). The red outline indicates the boundaries of the Great Plains Region (GPR).
