# Supplementary material for: Climate‐change‐driven shifts in C3 and C4 grass distributions and leaf traits could lead to changes in community‐level flammability
Source: Am J Bot. 2025 Aug 8;112(10):e70081. doi: 10.1002/ajb2.70081 (PMC12572686; doi:10.1002/ajb2.70081)
Supplement: Supplementary file 5 — Appendix S5. Pairwise Pearson correlation coefficients between habitat suitability projections across GCMs. [file AJB2-112-e70081-s014.pdf]

Appendix S5. Pairwise Pearson correlation coefficients (*r*) between habitat suitability projections across GCMs

Table S5. Pairwise Pearson correlation coefficients (*r*) between delta habitat suitability maps created with species distribution models (MaxEnt) across global climate models (GCMs), for both C3 and C4 grass species under SSP370 and SSP585 scenarios. Correlation values are high (r > 0.8) across all comparisons, indicating strong agreement in spatial patterns of projected suitability change. Given this consistency, we present results from only one representative model (MIROC6 SSP5 8.6) in the main text, with full model comparisons provided in the Supporting Information.

Table S5: Pairwise Pearson correlation coefficients (*r*) between habitat suitability projections across GCMs

Functional type	SSP scenario	Model pair	*r*
C_4	SSP3 7.0	ACCESS-CM2 vs CMCC-ESM-2-0	0.99
		ACCESS-CM2 vs EC-Earth3-Veg	0.84
		ACCESS-CM2 vs MIROC6	0.86
		CMCC-ESM-2-0 vs EC-Earth3-Veg	0.80
		CMCC-ESM-2-0 vs MIROC6	0.82
		EC-Earth3-Veg vs MIROC6	0.94
	SSP5 8.5	ACCESS-CM2 vs CMCC-ESM-2-0	1.00
		ACCESS-CM2 vs EC-Earth3-Veg	0.88
		ACCESS-CM2 vs MIROC6	0.89
		CMCC-ESM-2-0 vs EC-Earth3-Veg	0.89
		CMCC-ESM-2-0 vs MIROC6	0.90
		EC-Earth3-Veg vs MIROC6	0.96
C_3	SSP3 7.0	ACCESS-CM2 vs CMCC-ESM-2-0	0.99
		ACCESS-CM2 vs EC-Earth3-Veg	0.90
		ACCESS-CM2 vs MIROC6	0.91
		CMCC-ESM-2-0 vs EC-Earth3-Veg	0.87
		CMCC-ESM-2-0 vs MIROC6	0.88
		EC-Earth3-Veg vs MIROC6	0.95
	SSP5 8.5	ACCESS-CM2 vs CMCC-ESM-2-0	1.00
		ACCESS-CM2 vs EC-Earth3-Veg	0.93
		ACCESS-CM2 vs MIROC6	0.93
		CMCC-ESM-2-0 vs EC-Earth3-Veg	0.94
		CMCC-ESM-2-0 vs MIROC6	0.94
		EC-Earth3-Veg vs MIROC6	0.97
