# Supplementary material for: Climate‐change‐driven shifts in C3 and C4 grass distributions and leaf traits could lead to changes in community‐level flammability
Source: Am J Bot. 2025 Aug 8;112(10):e70081. doi: 10.1002/ajb2.70081 (PMC12572686; doi:10.1002/ajb2.70081)
Supplement: Supplementary file 6 — Appendix S6. Habitat suitability: summary of linear mixed‐effects model results for C4 and C3 species. [file AJB2-112-e70081-s009.pdf]

## Appendix S6. Habitat suitability: Summary of linear mixed-effects model results $C_4$-$C_3$

**Table S6.** Summary of linear mixed-effects model (lme4) results comparing plant type ($C_4$ and $C_3$) and scenario effects (future vs. present) on habitat suitability in North America. The table presents the fixed effects estimates from the linear mixed-effects model, including the intercept, main effects of plant type ($C_3$ vs. $C_4$), scenario (ambient vs. future), and their interaction. Post hoc pairwise comparisons between different plant type and scenario combinations are also included, showing the estimated differences, standard errors, *z* values, and *P*-values. Significant differences ($P < 0.05$) are highlighted, indicating how plant responses vary between ambient and future scenarios and between $C_3$ and $C_4$ species. Random effects variance for species and residuals are provided.

**Table S6a: Summary of linear mixed-effects model results $C_4$-$C_3$**

| **Effect** | **Est.** | **SE** | ***t*** | ***P*** |
|---|---|---|---|---|
| **Fixed effects** | | | | |
| Intercept ($C_3$ present) | 0.129 | 0.026 | 4.86 | <0.0001 |
| Plant type ($C_4$ vs. $C_3$) | 0.103 | 0.035 | 2.94 | 0.0176 |
| Scenario (Future vs. present | -0.053 | 0.001 | -66.27 | <0.0001 |
| Plant type x scenario interaction | 0.018 | 0.001 | 16.6 | <0.0001 |
| **Random effects**: | | | | |
| Species variance (intercept): 0.0112 | | | | |
| Residual variance: 0.0208 | | | | |

**Table S6b: Post hoc pairwise comparisons**

| **Contrast** | **Est.** | **SE** | ***z*** | ***P*** |
|---|---|---|---|---|
| $C_3$ Present - $C_4$ Present | -0.103 | 0.035 | -2.94 | 0.0176 |
| $C_3$ Present - $C_3$ Future | 0.053 | 0.001 | 66.27 | <0.0001 |
| $C_4$ Present - $C_3$ Future | 0.156 | 0.035 | 4.45 | 0.0001 |
| $C_4$ Present - $C_4$ Future | 0.035 | 0.001 | 50.69 | <0.0001 |
| $C_3$ Future - $C_4$ Future | -0.121 | 0.035 | -3.44 | 0.0033 |
