# Supplementary material for: Climate‐change‐driven shifts in C3 and C4 grass distributions and leaf traits could lead to changes in community‐level flammability
Source: Am J Bot. 2025 Aug 8;112(10):e70081. doi: 10.1002/ajb2.70081 (PMC12572686; doi:10.1002/ajb2.70081)
Supplement: Supplementary file 7 — Appendix S7. Species‐specific habitat suitability: summary of linear model results. [file AJB2-112-e70081-s007.pdf]

Appendix S7. Species-specific habitat suitability: Summary of linear model results

Table S7. Summary of linear model (lm) results comparing scenario effects (future vs. present) on the habitat suitability for each studied species. Significant differences ($P < 0.05$) are highlighted, indicating how each species is predicted to vary between ambient and future scenarios.

**Table S7: Summary of linear model results for each species**					
**Difference between predicted present and future**					
**C_4 species**	**Est.**	**SE**	***t***	***P***	**Δ Direction**
Andropogon gerardi	0.01	0.003	3.8	<0.0001	↑
Bouteloua gracilis	-0.15	0.003	-44.55	<0.0001	↓
Schizachyrium scoparium	-0.04	0.004	-11.55	<0.0001	↓
Sorghastrum nutans	0	0.003	-1.18	<0.0001	–
Andropogon hallii	0.03	0.005	4.83	<0.0001	↑
Aristida purpurea	0.08	0.004	19.96	<0.0001	↑
Bouteloua curtipendula	-0.03	0.003	-10.14	<0.0001	↓
Bouteloua dactyloides	-0.03	0.004	-7.57	<0.0001	↓
Bouteloua hirsuta	0.01	0.003	1.77	0.08	↑
Eragrostis trichodes	-0.1	0.004	-22.64	<0.0001	↓
Hilaria jamesii	0	0.003	-1.16	0.24	–
Muhlenbergia cuspidata	-0.18	0.004	-49.2	<0.0001	↓
Muhlenbergia filiformis	0	0.001	-6	<0.0001	–
Muhlenbergia reverchonii	-0.01	0.002	-6.94	<0.0001	↓
Panicum capillare	-0.08	0.003	-24	<0.0001	↓
Panicum virgatum	-0.02	0.004	-4.55	<0.0001	↓
Pappophorum bicolor	0.06	0.003	22.12	<0.0001	↑
Paspalum setaceum	-0.01	0.003	-4.04	<0.0001	↓
Sporobolus compositus	-0.16	0.004	-38.91	<0.0001	↓
Sporobolus heterolepis	-0.07	0.002	-41.74	<0.0001	↓
Tripsacum dactyloides	-0.03	0.002	-12.38	<0.0001	↓
**C_3 species**					
Agropyron repens	-0.02	0.002	-12.15	<0.0001	↓
Bromus inermis	-0.09	0.005	-16.78	<0.0001	↓
Koeleria cristata	-0.04	0.002	-19.83	<0.0001	↓
Poa pratensis	-0.12	0.003	-44.59	<0.0001	↓
Achnatherum robustum	-0.06	0.003	-19.66	<0.0001	↓
Agrostis scabra	-0.04	0.002	-24.66	<0.0001	↓
Alopecurus carolinianus	-0.02	0.003	-6.78	<0.0001	↓
Danthonia spicata	-0.01	0.001	-23.85	<0.0001	↓
Elymus elymoides	-0.05	0.002	-20.09	<0.0001	↓
Elymus villosus	-0.05	0.002	-21.46	<0.0001	↓
Festuca altaica	0	0	-10.22	<0.0001	↑
Hesperostipa comata	-0.08	0.004	-21.19	<0.0001	↓
Hesperostipa spartea	-0.08	0.003	-26.15	<0.0001	↓

Nassella leucotricha	0.01	0.003	2.7	<0.01	↑
Nassella viridula	-0.13	0.004	-36.94	<0.0001	↓
Poa palustris	-0.06	0.002	-26.93	<0.0001	↓
