# Supplementary material for: Climate‐change‐driven shifts in C3 and C4 grass distributions and leaf traits could lead to changes in community‐level flammability
Source: Am J Bot. 2025 Aug 8;112(10):e70081. doi: 10.1002/ajb2.70081 (PMC12572686; doi:10.1002/ajb2.70081)
Supplement: Supplementary file 8 — Appendix S8. Community composition: summary of linear mixed‐effects model results for C4 and C3 species. [file AJB2-112-e70081-s002.pdf]

Appendix S8. Community composition: Summary of linear mixed-effects model results C_4 and C_3

Table S8. Summary of linear mixed-effects model (lme4) results comparing plant type (C_4 and C_3) and scenario effects (future vs. present) on relative community composition in the Great Plains Region. The table presents the fixed effects estimates from the linear mixed-effects model, including the intercept, main effects of plant type (C_3 vs. C_4), scenario (ambient vs. future), and their interaction. Post hoc pairwise comparisons between different plant type and scenario combinations are also included, showing the estimated differences, standard errors, *z*-values, and *P*-values. Significant differences ($P < 0.05$) are highlighted, indicating how plant responses vary between ambient and future scenarios and between C_3 and C_4 species. Random effects variance for species and residuals are provided.

Table S8a: Summary of linear mixed-effects model results

Effect	Est.	SE	*t*	*P*
**Fixed effects**				
Intercept (C_3 Present)	0.02	0	3.83	<0.0001
Plant type (C_4 vs. C_3)	0.02	0.01	2.42	0.09
Scenario (Future vs. Present)	0	0	-1.75	0.09
Plant type x Scenario interaction	0.01	0	2.3	<0.05
**Random effects**:				

Species Variance (Intercept): 0.0003

Residual Variance: 0.00005

Table S8b: Post hoc pairwise comparisons

Contrast	Est.	SE	*z*	*P*
C_3 Present - C_4 Present	-0.02	0.01	-2.42	0.09
C_3 Present - C_3 Future	0	0	1.75	0.32
C_3 Present - C_4 Future	-0.02	0.01	-2.96	<0.05
C_4 Present - C_3 Future	0.02	0.01	3.17	<0.05
C_4 Present - C_4 Future	0	0	-1.49	0.46
