# Supplementary material for: Climate‐change‐driven shifts in C3 and C4 grass distributions and leaf traits could lead to changes in community‐level flammability
Source: Am J Bot. 2025 Aug 8;112(10):e70081. doi: 10.1002/ajb2.70081 (PMC12572686; doi:10.1002/ajb2.70081)
Supplement: Supplementary file 9 — Appendix S9. Predicted rate of fire spread: summary of linear mixed‐effects model results. [file AJB2-112-e70081-s011.pdf]

Raubenheimer et al. -American Journal of Botany 2025- Appendix S9.

Appendix S9 Predicted rate of fire spread: Summary of linear mixed-effects model results

Table S9: Summary of linear mixed-effects model (lme4) results comparing scenario effects [Present, Future + eCO_2 (Future1), and Future $+CO_2$, +N, +T, and $-H_20$ (Future2)] on community-level predicted rates of fire spread (ROS; Rothermel fire model) in the Great Plains Region. The table presents fixed effects estimates from the linear mixed-effects model, including the intercept for the Present scenario and the effects of Future1 and Future2 scenarios. Post hoc pairwise comparisons between scenarios are included, showing the estimated differences, standard errors, *z*-values, and *P*-values. Significant differences ($P < 0.05$) are highlighted, indicating how community values differ between the Present and Future scenarios. Random effects variance for *x*- and *y*-coordinates, as well as residual variance, are also provided.

Table S9a: Summary of linear mixed effects model results

**Effect**	**Est.**	**SE**	***t***	***P***
**Fixed Effects:**				
Intercept (Present)	42.092	0.666	63.22	<0.0001
ScenarioFuture1 (Future1)	-5.377	0.011	-468.37	<0.0001
ScenarioFuture2 (Future2)	-2.478	0.011	-215.87	<0.0001
**Random Effects:**	**Variance**	**S.D.**		
y (Intercept)	140.39	11.849		
x (Intercept)	368.27	19.19		
Residual	75.27	8.676		

Table S9b: Post hoc pairwise comparisons

**Contrast**	**Est.**	**SE**	***z***	***P***
Present - Future1	5.377	0.0115	468.37	<0.0001
Present - Future2	2.478	0.0115	215.87	<0.0001
Future1 - Future2	-2.899	0.0115	-252.5	<0.0001
