# Supplementary material for: Climate‐change‐driven shifts in C3 and C4 grass distributions and leaf traits could lead to changes in community‐level flammability
Source: Am J Bot. 2025 Aug 8;112(10):e70081. doi: 10.1002/ajb2.70081 (PMC12572686; doi:10.1002/ajb2.70081)
Supplement: Supplementary file 10 — Appendix S10. Habitat suitability: effect of bioclimatic variables (C3 and C4). [file AJB2-112-e70081-s008.pdf]

Appendix S10. Habitat suitability: Effect of bioclimatic variables. C_3 and C_4

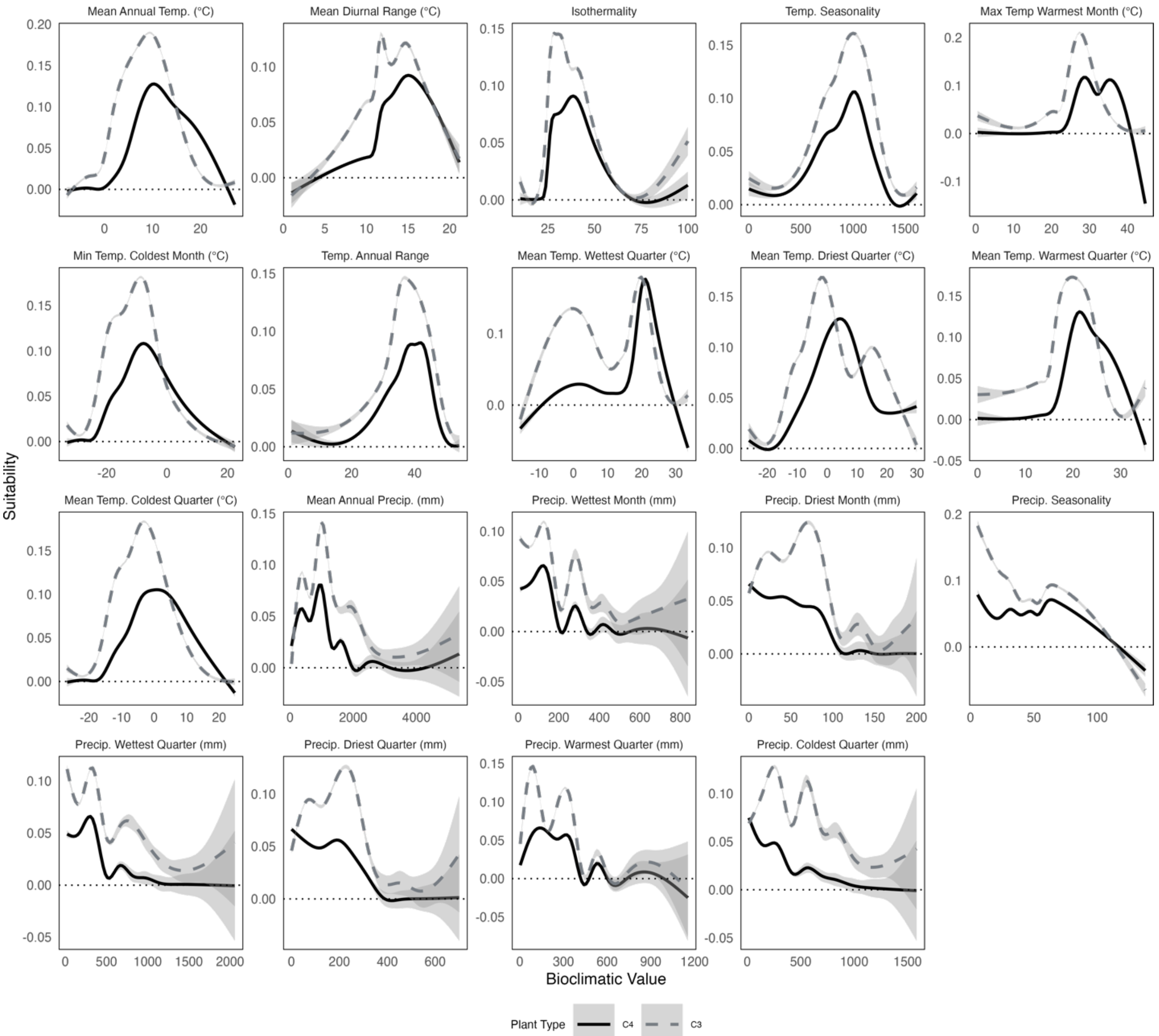

Figure S10. Relationship between bioclimatic variables and habitat suitability for C_4 (black, solid) and C_3 (gray, dashed) plant types. The figure displays smoothed curves representing the suitability of different plant types (C_3 and C_4) as a function of various bioclimatic variables. Each panel corresponds to a specific bioclimatic variable, with the *x*-axis showing the range of values for that variable and the *y*-axis indicating the predicted habitat suitability. Dotted horizontal lines at $y = 0$ represent the threshold for suitability.
