# Supplementary material for: Climate‐change‐driven shifts in C3 and C4 grass distributions and leaf traits could lead to changes in community‐level flammability
Source: Am J Bot. 2025 Aug 8;112(10):e70081. doi: 10.1002/ajb2.70081 (PMC12572686; doi:10.1002/ajb2.70081)
Supplement: Supplementary file 12 — Appendix S12. Species‐specific habitat suitability (C3). [file AJB2-112-e70081-s001.pdf]

Appendix S12. Species-specific habitat suitability (C_3)

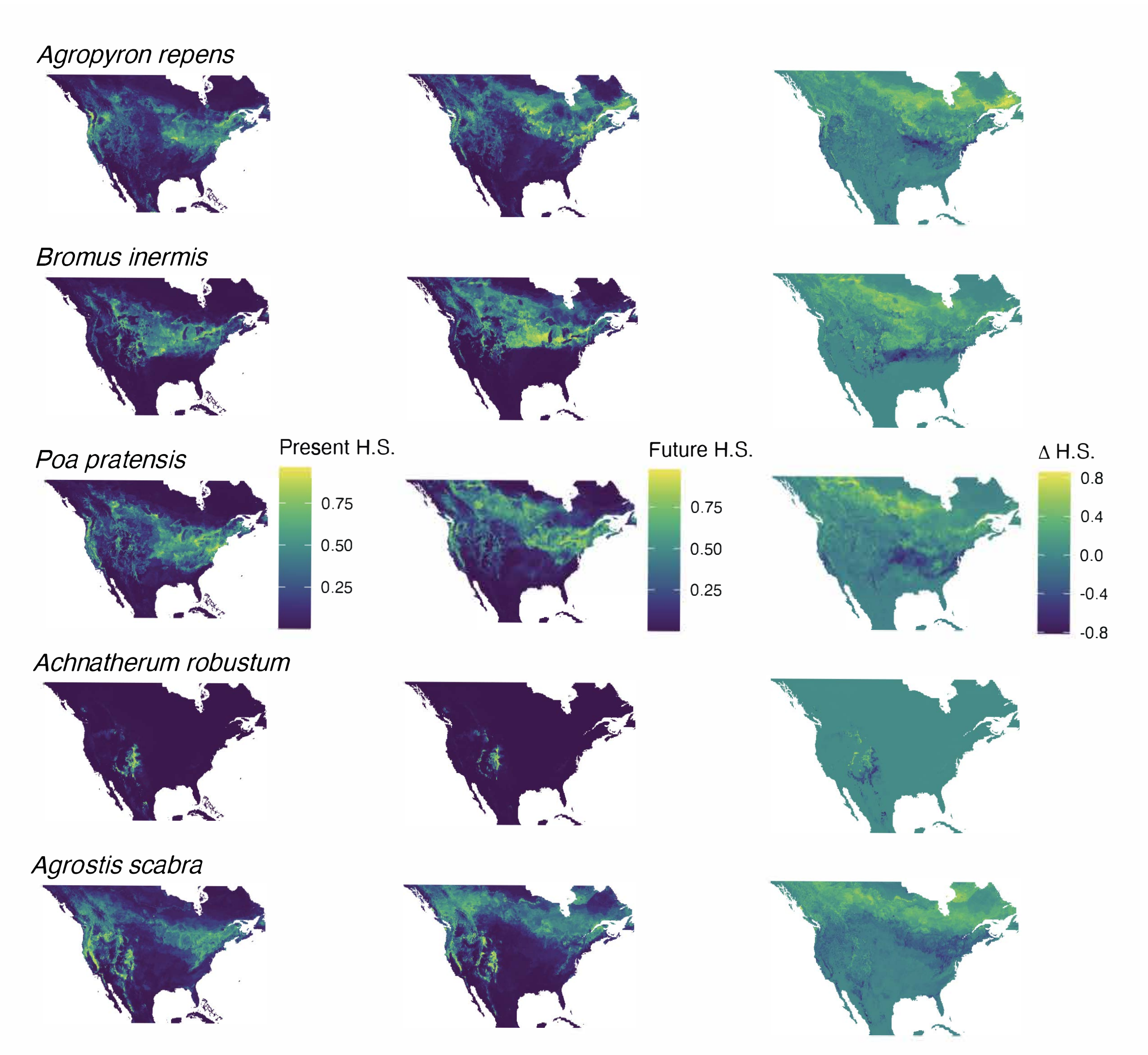

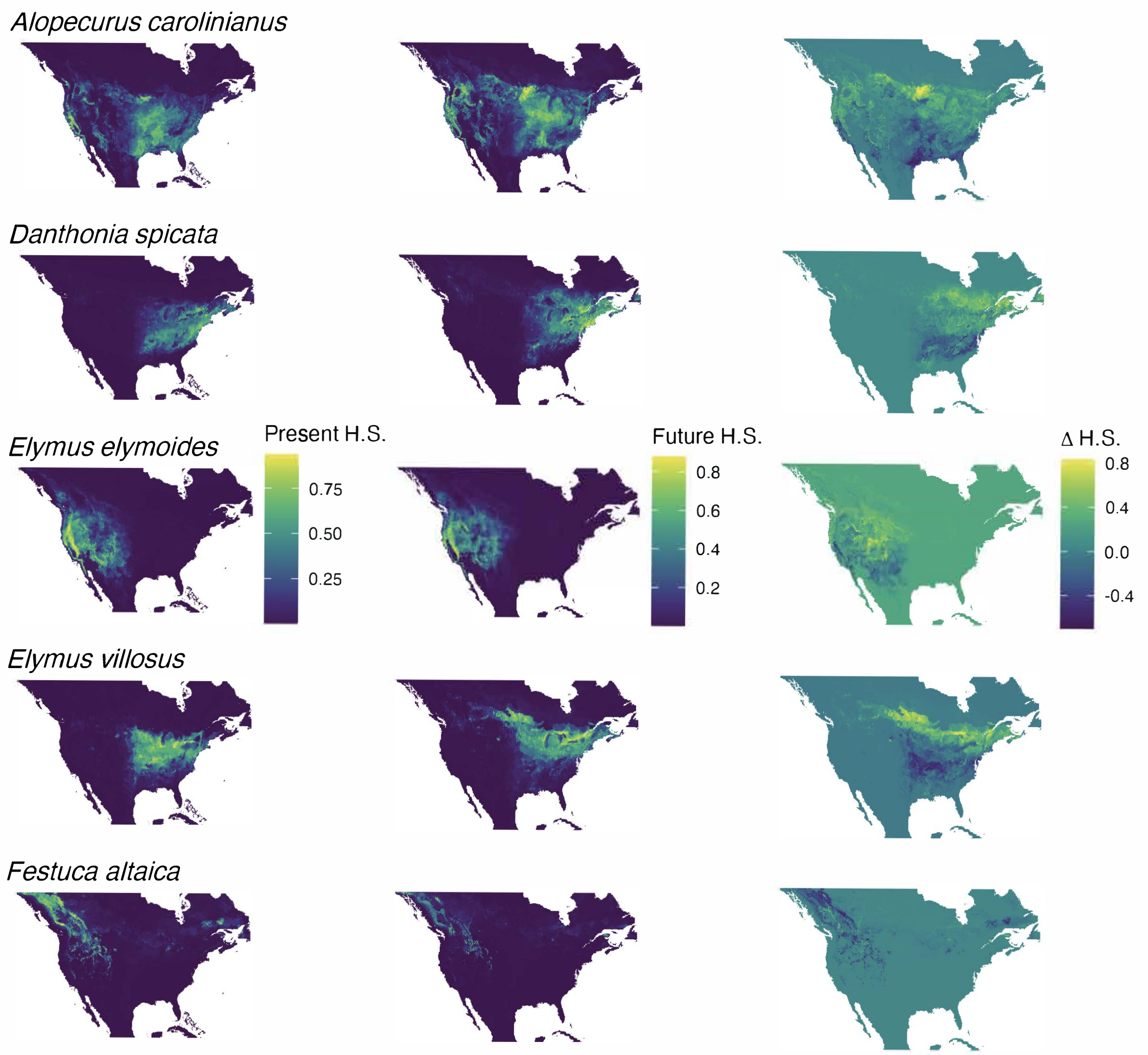
Alopecurus carolinianus
Danthonia spicata
Elymus elymoides
Present H.S.
0.75
0.50
0.25
Future H.S.
0.8
0.6
0.4
0.2
Δ H.S.
0.8
0.4
0.0
-0.4
Elymus villosus
Festuca altaica

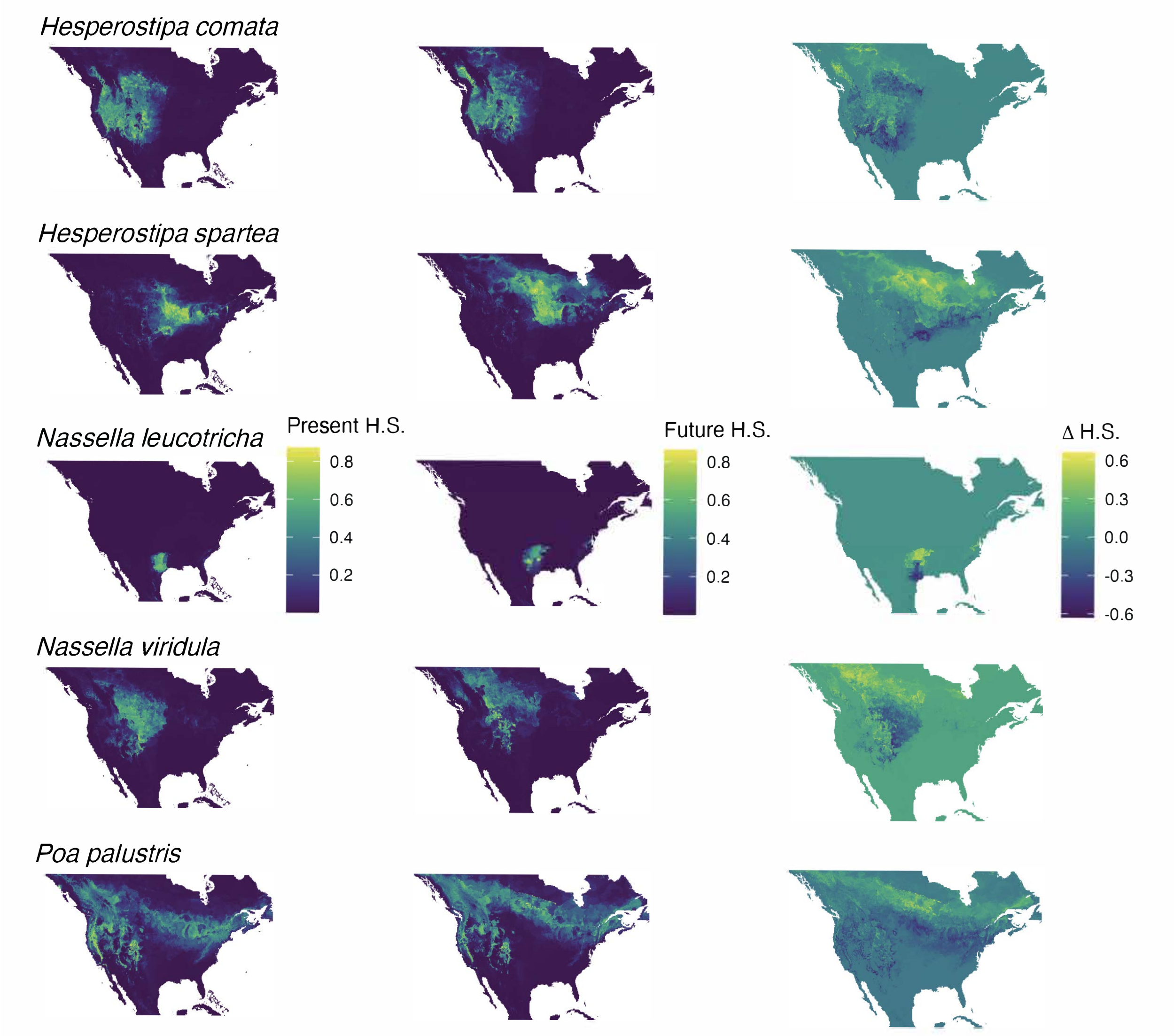

Figure S12. Changes in habitat suitability (H.S.) for selected C_3 grass species characteristic of the Great Plains region. Panel A represents current conditions, while B depicts projected conditions for 2060 (MIROC RCP 6.5). Panel C shows the difference between future and current suitability (ΔH.S.).
