# Supplementary material for: Climate‐change‐driven shifts in C3 and C4 grass distributions and leaf traits could lead to changes in community‐level flammability
Source: Am J Bot. 2025 Aug 8;112(10):e70081. doi: 10.1002/ajb2.70081 (PMC12572686; doi:10.1002/ajb2.70081)
Supplement: Supplementary file 13 — Appendix S13. Traits: summary of linear mixed‐effects model results. [file AJB2-112-e70081-s013.pdf]

Raubenheimer et al. -American Journal of Botany 2025- Appendix S13.

Appendix S13. Traits: Summary of linear mixed-effects model results

Table S13. Results of linear mixed-effects models analyzing the effects of elevated CO_2 and combined environmental treatments (+CO_2, +N, +T, -H_2O) on various traits in C_3 and C_4 grass species. The table includes estimates (Est.), standard errors (SE), *t*-values, and *P*-values for treatment effects and species-type interactions on aboveground biomass (AG biomass), carbon assimilation rate (*A*) leaf water content (LWC), gas exchange (Gst), specific leaf area (SLA), and water-use efficiency (WUE).

Table S13: Summary of linear mixed-effects model results

**Trait**	**Effect**	**Est.**		***t***	***P***
	**Fixed effects**				
	Intercept (C_3 Present)	17.54	3.74	4.69	<0.0001
	Treatment: + CO_2	-17.26	3.89	-4.44	<0.01
	Treatment: + CO_2+N +T - H_2O	-29.41	6	-4.9	<0.01
	Type: C_4	27.09	7.87	3.44	<0.01
AG biomass	Treatment: + CO_2* Type: C_4	-37.79	9.01	-4.19	<0.001
	Treatment: + CO_2+N +T - H_2O * Type: C_4	-62.87	9.49	-6.62	<0.0001
	**Random effects:**				
	**Variance component**				
	Plot variance (Intercept)	2.34			
	Species variance (Intercept)	0.82			
	Residual variance	1.21			
	**Fixed effects**				
	Intercept (C_3 Present)	6.44	2.22	2.91	<0.01
	Treatment: + CO_2	4.68	3.79	1.24	0.25
	Treatment: + CO_2+N +T - H_2O	5.87	3.66	1.6	0.15
	Type: C4	2.53	3.09	0.82	0.42
A	Treatment: + CO_2* Type: C_4	2.92	5.11	0.57	0.57
	Treatment: + CO_2+N +T - H_2O * Type: C_4	0.63	5.3	0.12	0.91
	**Random effects:**				
	**Variance component**				
	Plot variance (Intercept)	1.76			
	Species variance (Intercept)	0.58			
	Residual variance	0.89			
	**Fixed effects**				
	Intercept (C_3 Present)	0.15	0.038	3.92	< 0.0001
	Treatment: + CO_2	-0.042	0.063	-0.67	0.52
	Treatment: + CO_2+N +T - H_2O	-0.069	0.061	-1.12	0.29
	Type: C_4	-0.063	0.044	-1.45	0.16
	Treatment: + CO_2* Type: C_4	0.113	0.071	1.58	0.13
LWC	Treatment: + CO_2+N +T - H_2O * Type:	0.096	0.074	1.29	0.21
	C_4 **Random effects:**				
	**Variance component**				
	Plot variance (Intercept)	0.09			
	Species variance (Intercept)	0.03			
	Residual variance	0.05			
G_{st}	**Fixed effects**				

	Intercept (C_3 Current)	0.056	0.013	4.36	< 0.0001
	Treatment: + CO_2	0.039	0.022	1.82	0.11
	Treatment: + CO_2+N +T - H_2O	0.026	0.021	1.22	0.26
	Type: C_4	0.031	0.014	2.19	0.04
	Treatment: + CO_2* Type: C_4	-0.033	0.024	-1.39	0.18
	Treatment: + CO_2+N +T - H_2O * Type: C_4	-0.037	0.025	-1.52	0.14
	**Random effects:**				
	**Variance component**				
	Plot variance (Intercept)	0.06			
	Species variance (Intercept)	0.02			
	Residual variance	0.03			
SLA	**Fixed effects**				
	Intercept (C_3 Present)	163.12	11.28	14.46	< 0.0001
	Treatment: + CO_2	39	17.4	2.24	0.06
	Treatment: + CO_2+N +T - H_2O	20.84	18.17	1.15	0.28
	Type: C_4	18.53	15.62	1.19	0.25
	Treatment: + CO_2* Type: C_4	-32.42	23.52	-1.38	0.18
	Treatment: + CO_2+N +T - H_2O * Type: C_4	0.75	23.8	0.03	0.98
	**Random effects:**				
	**Variance component**				
	Plot variance (Intercept)	1.21			
	Species variance (Intercept)	0.54			
	Residual variance	0.61			
WUE	**Fixed effects**				
	Intercept (C_3 Present)	122.08	19.25	6.34	< 0.0001
	Treatment: + CO_2	-0.95	32.96	-0.03	0.98
	Treatment: + CO_2+N +T -H_2O	18	31.84	0.57	0.59
	Type: C4	-3.82	22.32	-0.17	0.87
	Treatment: + CO_2* Type: C_4	56.12	37.47	1.5	0.15
	Treatment: + CO_2+N +T - H_2O * Type: C_4	50.69	38.43	1.32	0.2
	**Random effects**:				
	**Effect**				
	Plot variance (Intercept)	24.66			
	Species variance (Intercept)	43.38			
	Residual variance	42.33			
