# Supplementary material for: Climate‐change‐driven shifts in C3 and C4 grass distributions and leaf traits could lead to changes in community‐level flammability
Source: Am J Bot. 2025 Aug 8;112(10):e70081. doi: 10.1002/ajb2.70081 (PMC12572686; doi:10.1002/ajb2.70081)
Supplement: Supplementary file 14 — Appendix S14. Species‐specific predicted rate of fire spread. [file AJB2-112-e70081-s010.pdf]

Appendix S14. Species-specific predicted rate of fire spread

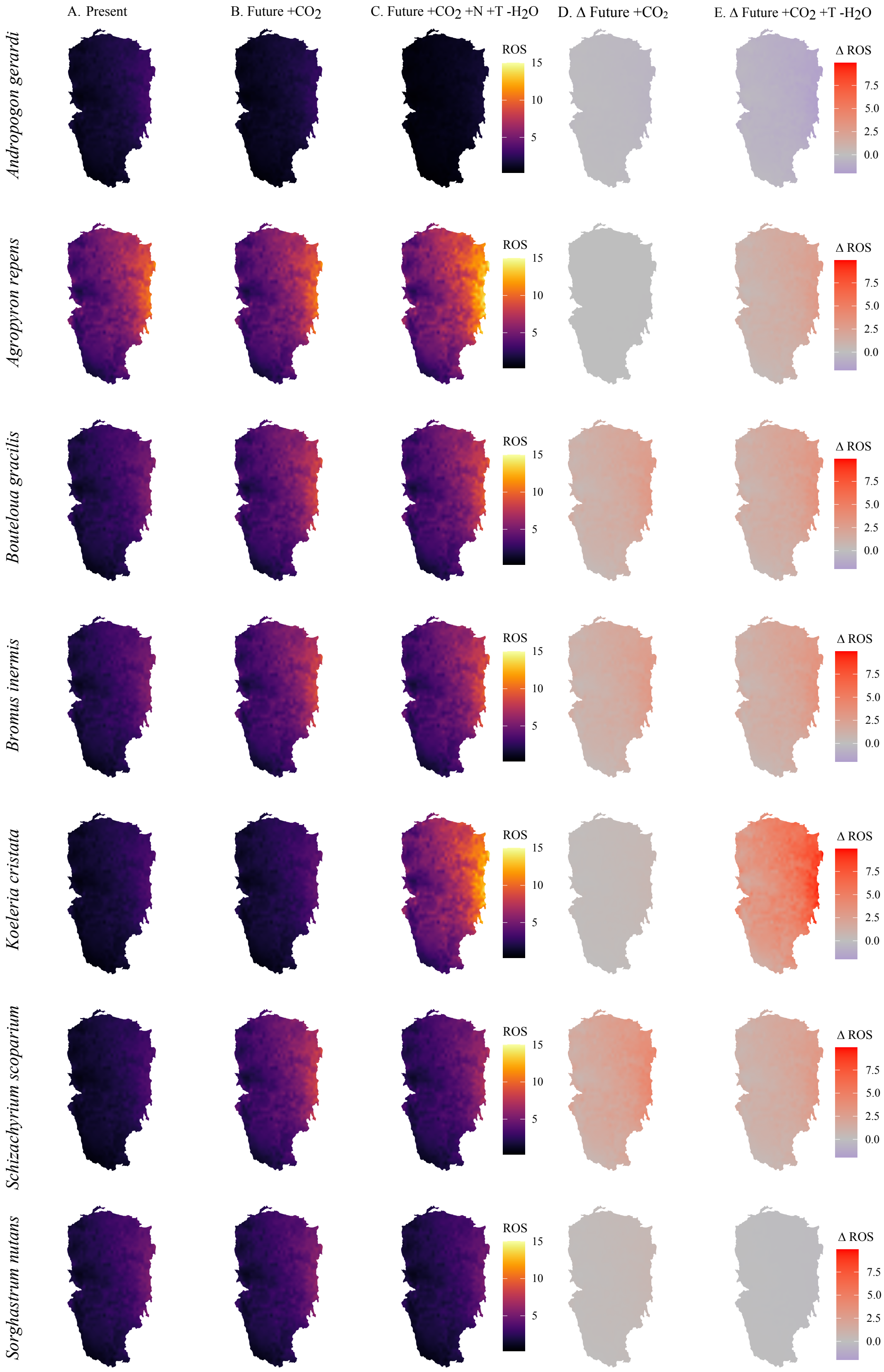

A. Present
B. Future +CO2
C. Future +CO2 +N +T -H2O
D. Δ Future +CO2
E. Δ Future +CO2 +T -H2O
Andropogon gerardi
Agropyron repens
Bouteloua gracilis
Bromus inermis
Koeleria cristata
Schizachyrium scoparium
Sorghastrum nutans
ROS
15
10
5
Δ ROS
7.5
5.0
2.5
0.0

Figure S14: Changes in the mean predicted rate of fire spread (ROS) for seven grass species from the BioCON experiment within the Great Plains region. Panel A shows present conditions; panel B presents future projections for 2060 (MIROC RCP 6.5), incorporating the effects of elevated CO_2 (+CO_2) on flammability-related leaf traits. Panel C further includes the effects of elevated nitrogen (+N), elevated temperature +T), and reduced water availability (-H_2O). Panels D and E illustrate the differences in ROS between ambient conditions and future scenarios, with panel D showing the impact of +CO_2 and panel E the combined effects of +CO_2, +N, +T, and -H_2O. Species included in the ROS models are *Andropogon gerardii, Schizachyrium scoparium, Sorghastrum nutans* (C_4 species), *Agropyron repens, Bromus inermis, Koeleria cristata,* and *Poa pratensis* (C_3 species).

Table S14: Estimated marginal mean (EMM) and pairwise comparisons for scenario effects within species for three scenarios—Present, Future + eCO2 (Future1), and Future +CO_2, +N, +T, and -H_20 (Future2)—across multiple grass species. The 95% confidence intervals (95% C.I.) are provided alongside each EMM. Pairwise comparisons between scenarios are detailed with contrast estimates (Est.), *t*-values, and associated *P*-values, highlighting significant differences in species responses to different future scenarios.

Table S14: Estimated marginal means and pairwise comparisons for scenario effects within species

**Species**	**Scenario**	**EMM**	**95% C.I.**	**Contrast**	**Est.**	***t***	***P***
	Present	50.33	50.30±50.35	Present - Future1	0.27	14.53	<0.0001
Andropogon gerardii	Future1	50.06	50.03±50.08	Present - Future2	-13.62	-730.18	<0.0001
	Future2	63.95	63.93±63.98	Future1 - Future2	-13.9	-744.71	<0.0001
	Present	17.02	16.99±17.04	Present - Future1	2.93	157.09	<0.0001
Agropyron repens	Future1	14.08	14.06±14.11	Present - Future2	8.53	457.34	<0.0001
	Future2	8.48	8.46±8.51	Future1 - Future2	5.6	300.25	<0.0001
	Present	26.99	26.96±27.01	Present - Future1	-14.49	-776.56	<0.0001
Bromis inermis	Future1	41.48	41.45±41.50	Present - Future2	-17.09	-916.1	<0.0001
	Future2	44.08	44.06±44.11	Future1 - Future2	-2.6	-139.55	<0.0001
	Present	26.99	26.96±27.01	Present - Future1	-14.49	-776.56	<0.0001
Koelaria cristata	Future1	41.48	41.45±41.50	Present - Future2	-17.09	-916.1	<0.0001
	Future2	44.08	44.06±44.11	Future1 - Future2	-2.6	-139.55	<0.0001
	Present	18.61	18.58±18.63	Present - Future1	-21.39	-1146.16	<0.0001
Schizachyrium scoparium	Future1	40	39.97±40.02	Present - Future2	-15.35	-822.83	<0.0001
	Future2	33.96	33.94±33.99	Future1 - Future2	6.03	323.33	<0.0001
	Present	25.34	25.32±25.37	Present - Future1	-3.05	-163.46	<0.0001
Sorghastrum nutans	Future1	28.39	28.37±28.42	Present - Future2	0.95	51.07	<0.0001
	Future2	24.39	24.36±24.41	Future1 - Future2	4	214.53	<0.0001
	Present	18.3	18.27±18.32	Present - Future1	-3.12	-167.46	<0.0001
Poa pratensis	Future1	21.42	21.39±21.45	Present - Future2	-42.72	-2289.65	<0.0001
	Future2	61.02	60.99±61.04	Future1 - Future2	-39.6	-2122.19	<0.0001
